# Activation of the DnaK-ClpB Complex is Regulated by the Properties of the Bound Substrate

**DOI:** 10.1038/s41598-018-24140-5

**Published:** 2018-04-11

**Authors:** Jose Angel Fernández-Higuero, Alejandra Aguado, Judit Perales-Calvo, Fernando Moro, Arturo Muga

**Affiliations:** Biofisika Institute (CSIC, UPV/EHU) and Department of Biochemistry and Molecular Biology, Faculty of Science and Technology, University of the Basque Country (UPV/EHU), P.O. Box 644, 48080 Bilbao, Spain

## Abstract

The chaperone ClpB in bacteria is responsible for the reactivation of aggregated proteins in collaboration with the DnaK system. Association of these chaperones at the aggregate surface stimulates ATP hydrolysis, which mediates substrate remodeling. However, a question that remains unanswered is whether the bichaperone complex can be selectively activated by substrates that require remodeling. We find that large aggregates or bulky, native-like substrates activates the complex, whereas a smaller, permanently unfolded protein or extended, short peptides fail to stimulate it. Our data also indicate that ClpB interacts differently with DnaK in the presence of aggregates or small peptides, displaying a higher affinity for aggregate-bound DnaK, and that DnaK-ClpB collaboration requires the coupled ATPase-dependent remodeling activities of both chaperones. Complex stimulation is mediated by residues at the β subdomain of DnaK substrate binding domain, which become accessible to the disaggregase when the lid is allosterically detached from the β subdomain. Complex activation also requires an active NBD2 and the integrity of the M domain-ring of ClpB. Disruption of the M-domain ring allows the unproductive stimulation of the DnaK-ClpB complex in solution. The ability of the DnaK-ClpB complex to discrimínate different substrate proteins might allow its activation when client proteins require remodeling.

## Introduction

The bacterial disaggregase ClpB collaborates with the Hsp70 (DnaK) system in the solubilization and reactivation of protein aggregates generated after exposure to harsh conditions^1–3^. Association of the disaggregase machinery to protein aggregates is a sequential reaction in which DnaJ binds first the aggregate and recruits DnaK, which finally drives the association of the disaggregase ClpB^4,5^. The functional oligomerization state of ClpB is the hexamer, which is built by identical subunits that fold into four domains: a N-terminal domain connected with the rest of the protein by a conserved linker, a first nucleotide binding domain (NBD1) in which the unique middle (M) domain is inserted, and a second NBD (NBD2)^6^. Both NBDs hydrolyze ATP, and must couple their ATPase activity to drive the unidirectional translocation of unfolded protein substrates through the axial protein channel^7,8^. The unique, flexible M domain is involved in the interaction of the disaggregase with DnaK^9–11^, stabilization of the hexameric particle^12^, and communication between adjacent subunits and between the two NBD tiers^13–16^. The position of the N-terminal and M domains relative to the barrel axis changes during the functional cycle of the chaperone, which is driven by nucleotide binding and hydrolysis at the NBDs^17,18^.

DnaK, as other members of the Hsp70 family, participates in diverse cellular functions, including protein folding, disaggregation and disassembly of specific complexes. This multifunctionality depends on the interaction with specific cochaperones (J proteins and nucleotide exchange factors) or collaborative chaperones (members of the Hsp60, Hsp90 or Hsp100 families)^19–21^. Engagement of DnaK in protein refolding requires its collaboration with DnaJ and GrpE whereas its role in aggregate disaggregation also needs ClpB^20,22,23^. DnaK undergoes a conformational cycle associated to its ATPase activity that regulates its interaction with substrates or cochaperone/chaperone partners. It has two domains connected by a conserved linker. The N-terminal, 40 kDa nucleotide binding domain (NBD) binds and hydrolyzes ATP, and the C-terminal, approx. 30 kDa domain interacts with protein substrates (SBD). The SBD can be further divided into two subdomains, the first 115 residues fold into a predominant β-structure and hold the peptide binding site (β-SBD), whereas the last 100 residues adopt a helical structure (α-SBD). Fine tuning of the intradomain allosteric communication driven by ATP hydrolysis and cochaperones^24^ controls the accessibility of the peptide binding site and the exposure of protein surfaces involved in the interaction with different partners. The allosterically-regulated, structural flexibility of the SBD allows the chaperone to bind and accommodate substrates of different sizes and conformational properties. When the substrate is a large, natively folded protein or a protein aggregate, the lid cannot close onto the β-sandwich, whereas it does when the client is a small peptide or a fully extended polypeptide^25–27^. These conformations might not be the only states that the SBD can adopt, and others with intermediate degrees of lid closure could exist depending on the properties of the bound substrates. The structural variability of the SBD also extends to the β-SBD, whose conformation can be similarly regulated by other protein regions, accessory proteins and substrates^28^.

ATP hydrolysis by both DnaK and ClpB and the threading activity of the disaggregase are critical for aggregate reactivation, and it has been proposed that the activation observed upon DnaK-ClpB complex formation is essential to regulate the function of this protein machinery^10,13^. Therefore, activation must be tightly regulated, as suggested by the fact that expression of permanently activated variants of ClpB is toxic for *E*. *coli*^10,13^. We further explore herein how stimulation of the DnaK-ClpB complex is affected by different substrate proteins. Our data show that the bichaperone complex is only stimulated in the presence of protein aggregates or bulky, native-like substrates that require remodeling, and not by a smaller, permanently unfolded proteins or short peptides. This substrate dependence might be a sophisticated way to regulate protein remodeling by this chaperone complex *in vivo*. Our data also show that activation of the bichaperone complex requires the coupled activity of both chaperones, as inactivation of any of them hampers formation of the stimulated complex. ClpB needs an active NBD2 and the integrity of the M domain ring for activation to occur. When its integrity is compromised, unproductive complex stimulation is observed in the absence of substrate. The DnaK region responsible for activation is located at the flexible β-SBD, where the substrate binding site is located, whose accessibility to ClpB is controlled by the allosteric displacement of the lid. The interaction between this DnaK region and ClpB could help substrate hand-off from DnaK to ClpB.

## Results

### Activation of the DnaK-ClpB complex depends on the protein substrate

**Figure 1:**
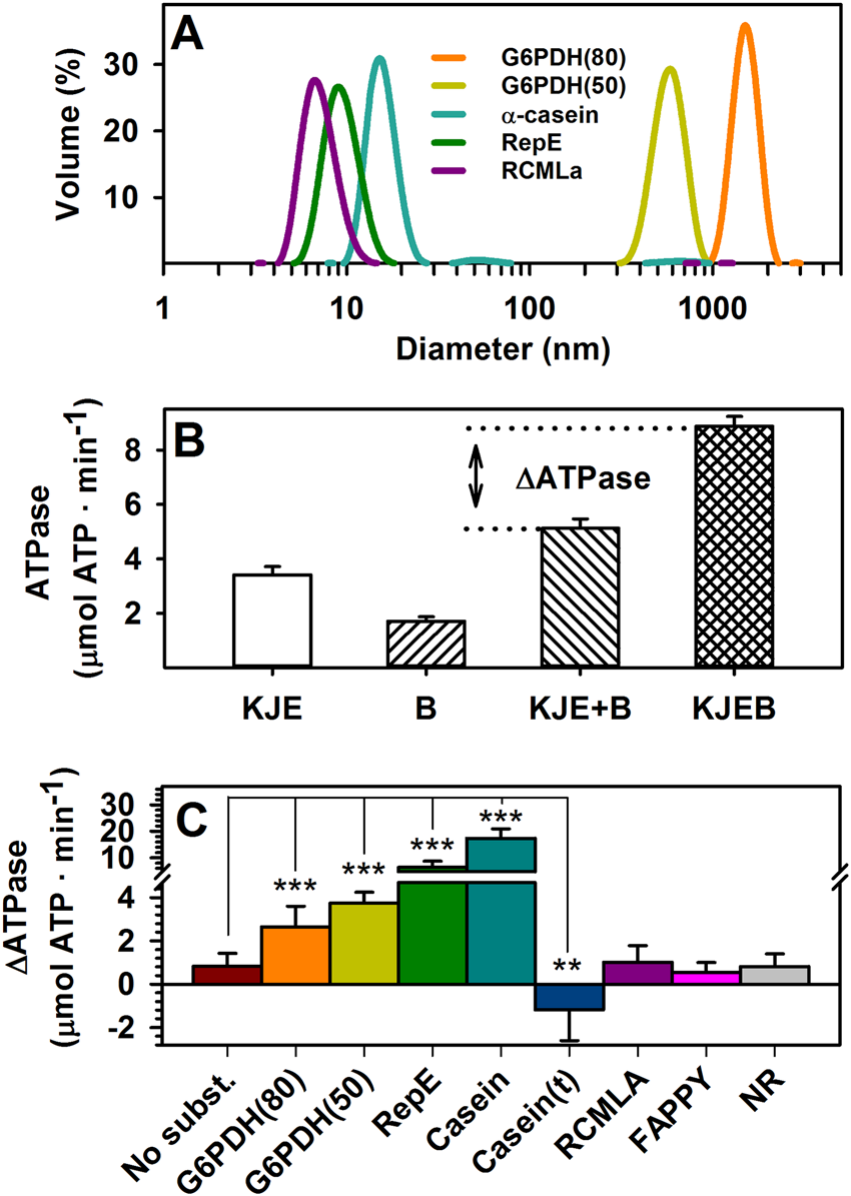
Activation of the DnaK-ClpB complex depends on the protein substrate. (**A**) Average size of aggregated and native substrates estimated by Dynamic Light Scattering (DLS). (**B**) ATPase activity of the DnaK system (KJE) and ClpB (**B**) measured independently in the presence of 0.4 μM G6PDH_50_, the arithmetic addition of both activities (KJE + B) and the experimental value obtained for the chaperone mixture (KJEB) under the same experimental conditions. (**C**) ATPase activity increase (ΔATPase), calculated as the difference between the experimental and expected activities, measured in the absence or presence of the following substrates: G6PDH_80_ or G6PDH_50_ aggregates (0.4 μM), RepE (5 µM), α-casein (10 µM), trypsinized α-casein (t) (10 µM casein treated with trypsin), RCMLA (10 µM), F-APPY (10 µM), and NR (350 µM). Data are shown as mean ± s.d. of n ≥ 3 independent experiments (**P < 0.01; ***P < 0.001).

DnaK and ClpB interact with a great variety of substrates, ranging from aggregates of variable size and conformation to native-like substrates or extended, short peptides. This interaction can stimulate both chaperones, which provide the energy needed for substrate remodeling. Several groups have also found an additional activation that depends on DnaK-ClpB complex formation, which becomes significantly stronger in the presence of an aggregated substrate^10,29,30^. However, the structural basis for this activation has not been analyzed in detail yet. To this aim, the stimulation of the ATPase activity of the bichaperone network due to complex formation was estimated in the presence of substrates that differ in size and conformational properties. These substrates include large aggregates of G6PDH formed at 50 (G6PDH_50_) or 80 °C (G6PDH_80_); three proteins, RepE, α-casein, and RCMLA, of different molecular masses and conformational properties^31^; and two short extended peptides, F-APPY and NR (22 and 7 amino acids long, respectively), which bind to DnaK with high affinity^32,33^. The hydrodynamic radius of G6PDH aggregates, α-casein, RepE and RCMLA was measured by DLS (Fig. 1A).

ATPase stimulation of the bichaperone mixture was estimated by, first, measuring independently the ATPase activity of the DnaK system (DnaK, DnaJ and GrpE) and ClpB in the absence or presence of the different substrates (Fig. 1B; and Supplementary Fig. S1 and Table S1). Then, these activities were arithmetically added to obtain the expected activity in the absence of a stimulatory action due to the interaction (KJE + B), and compared with the experimental value measured for the mixture of chaperones under identical conditions (KJEB) (Fig. 1; Supplementary Fig. S1 and Table S1). The increase in the ATPase activity obtained from the subtraction of these values was used to compare the activation of the bichaperone complex in the presence of the aforementioned protein substrates (Fig. 1C and Supplementary Fig. S1). A slight activation was observed in the absence of substrates, whereas in complex with both protein aggregates the stimulation was stronger, as previously reported^10,29,30^ (Fig. 1C and Supplementary Fig. S1). Smaller substrates exerted different effects: while RepE and α-casein displayed the strongest activation of the bichaperone complex, the stimulation in the presence of RCMLA or the short peptides F-APPY and NR was comparable to that observed in the absence of substrates (Fig. 1C and Supplementary Fig. S1). The higher ΔATPase values obtained in the presence of RepE and casein are expected as they are used at saturating concentrations, in contrast to protein aggregates. Interestingly, tryptic fragments of casein smaller than 10 kDa (Supplementary Fig. S2) failed to stimulate the DnaK-ClpB complex, in contrast to the full-length substrate (Fig. 1C and Supplementary Fig. S1), suggesting that activation is independent of specific peptide sequences. However, the possibility that sequences including or surrounding the trypsin cleavage sites might be responsible for the activation cannot be ruled out. Activation of the DnaK/ClpB complex was observed when either both chaperones were independently activated (casein and RepE) or when none of them were stimulated (G6PDH aggregates) (Supplementary Fig. S1). On the other hand, failure to form an activated chaperone complex occurred when both were independently and similarly activated (F-APPY, RCMLA and NR) (Supplementary Fig. S1 and Table S1). These data suggest that functional coupling of both chaperones might depend on specific properties of the client protein they interact with.

**Figure 2:**
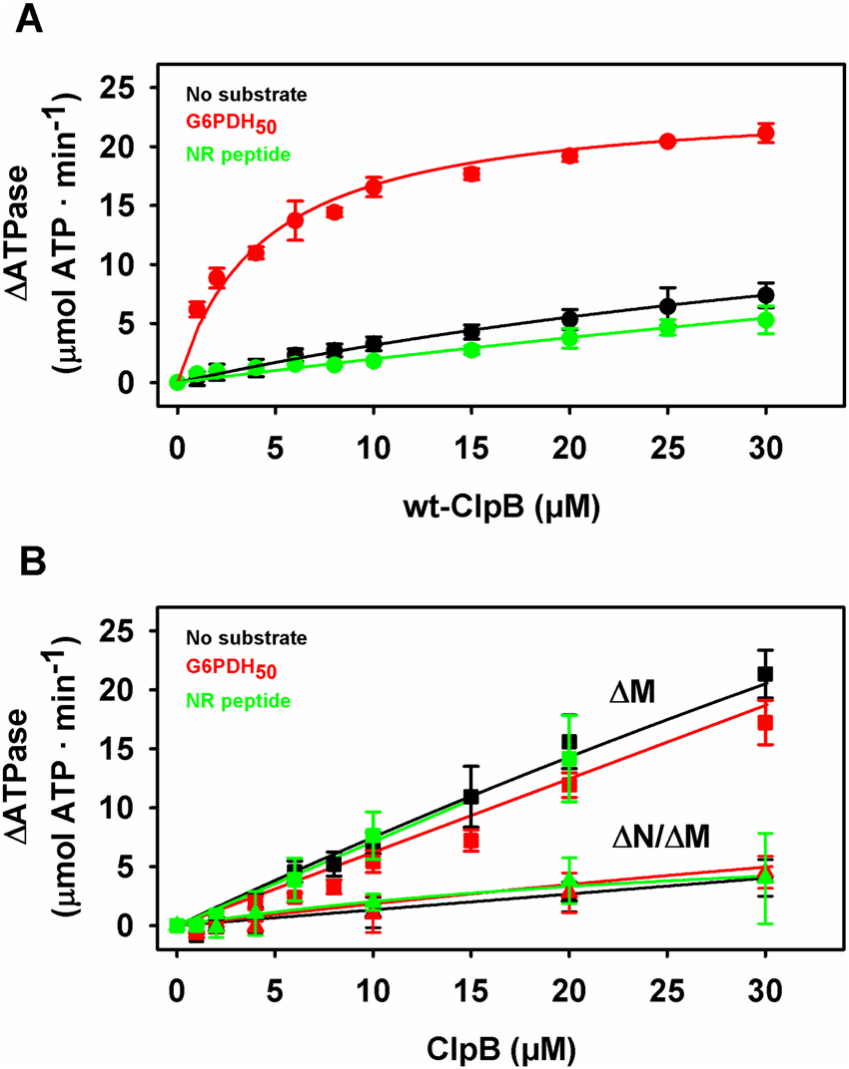
The affinity of ClpB for aggregate-bound DnaK is higher than for DnaK-peptide complexes. Stimulation of the DnaK-ClpB complex at increasing concentrations of (**A**) wt ClpB; (**B**) ΔM-ClpB or **ΔN/**ΔM-ClpB in the absence of substrates (black symbols), and in the presence of 1.2 μM aggregated G6PDH (red) or 350 μM NR peptide (green). The higher aggregate concentration (1.2 µM G6PDH_50_) used in these experiments favors association of the chaperones to the aggregate and thus results in a higher activation. ΔATPase values were calculated as the difference between the experimental activity of the bichaperone mixture and the activity of both KJE and ClpB, measured independently under the same experimental conditions. The K_0.5_ value obtained for wt-ClpB in the presence of aggregates was 4 ± 1 μM. Data are presented as mean ± s.d. of at least three independent experiments.

We next titrated DnaK with increasing ClpB concentrations in the absence and presence of distinct substrates, i.e., G6PDH_50_ aggregates and the NR peptide, to estimate the apparent affinity of the disaggregase for DnaK (Fig. 2 and Supplementary Table S2). In the absence of protein substrates and in the presence of the NR peptide, the apparent affinity was too low to be accurately estimated from the experimental data (Fig. 2A), in agreement with published data^11^. In contrast, the 5–10 times stronger activation observed in the presence of protein aggregates saturated with an estimated K_0.5_ value of 4.1 μM. These data indicate that both chaperones interact with a remarkable higher affinity in the presence of aggregates. As binding to client proteins unleashes the conformational plasticity of DnaK SBD^25,27,28^, activation of the bichaperone complex might depend on the interaction of the disaggregase with the varied conformations that DnaK expectedly adopts in complex with distinct client proteins.

### Interplay between the subdomains of the DnaK SBD regulates the activation of the bichaperone complex

**Figure 3:**
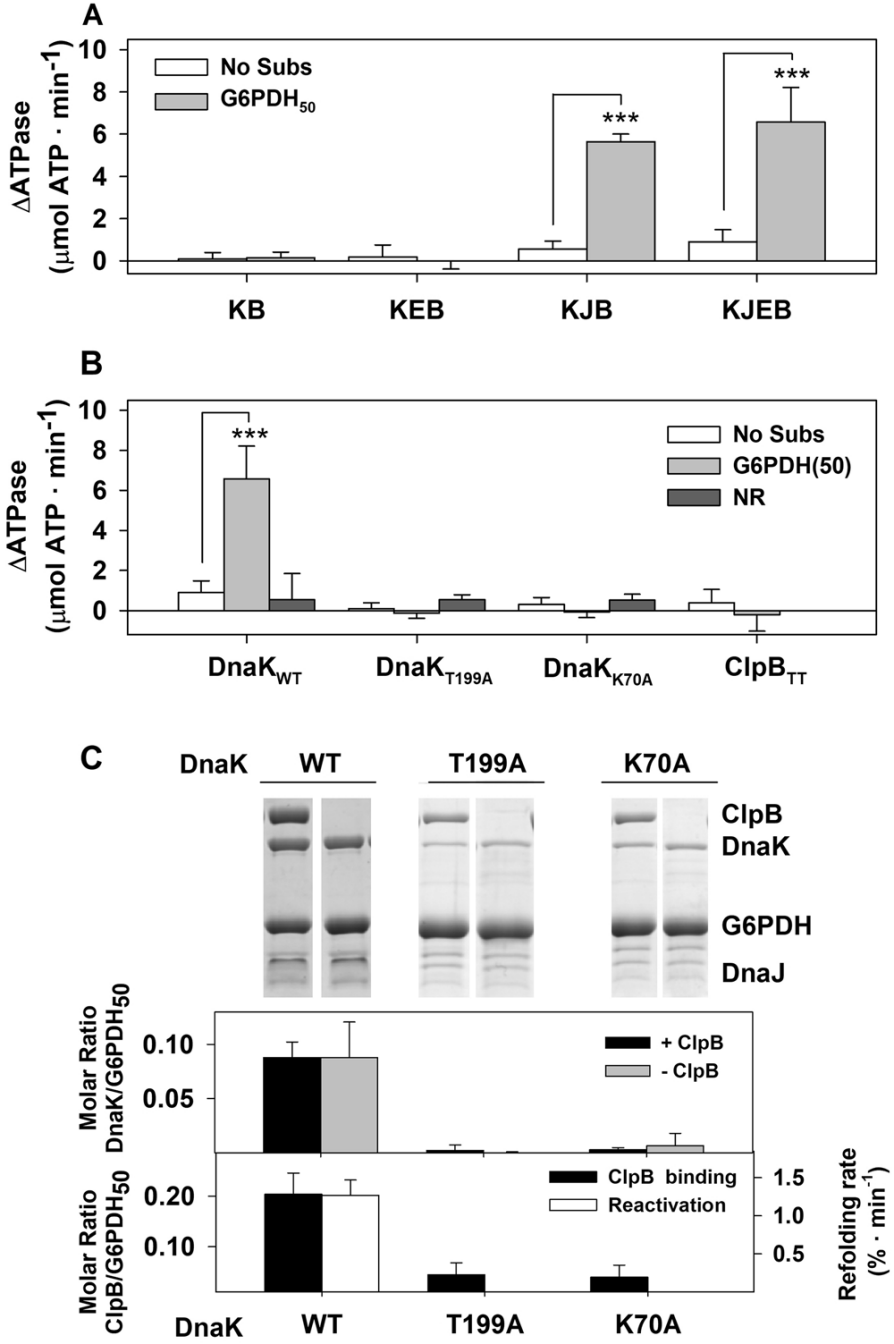
The ATPase activities of both chaperones are required to generate an activated and productive bichaperone complex. (**A**) Stimulation of the ClpB-DnaK complex by different cochaperone combinations in the absence and presence of 1.2 µM G6PDH_50_. (**B**) Activation of the bichaperone complex using inactive mutants of DnaK (DnaK_T199A_ and DnaK_K70A_) and ClpB (ClpB_TT_). (**C**) Binding of wt-DnaK and the inactive chaperone variants DnaK_T199A_ and DnaK_K70A_ to aggregates in the absence and presence of ClpB. G6PDH_50_ aggregates were diluted to 1 μM in refolding buffer containing 3 mM ATP, DnaK (3.5 μM), DnaJ (0.7 μM) without GrpE to slow down aggregate reactivation. When present, ClpB concentration was 5 μM. Samples were incubated 10 min and centrifuged to separate free and aggregate-bound chaperones. The resulting pellets were analyzed by 7.5% SDS-PAGE (upper panel) to estimate the amount of aggregate-bound DnaK (middle panel) or ClpB (lower panel), using known concentrations of the proteins as standards. Data are shown as mean ± s.d. of three independent experiments (***P < 0.001).

To explore the components of the DnaK system that are involved in the stimulation of the bichaperone complex at the aggregate surface, we tested whether DnaK alone or with different combinations of its cochaperones could form an activated complex with ClpB in the presence of protein aggregates. Data clearly showed that the activated complex could be generated only in the presence of DnaJ (Fig. 3A and Supplementary Table S3). We next sought to analyze the contribution of each chaperone to the stimulation observed for the aggregate-bound bichaperone complex. To this aim, we used two DnaK variants (DnaK_K70A_ and DnaK_T199A_) that have impaired ATP hydrolysis and the inactive ClpB_TT_ mutant, in which the conserved glutamic residues at the Walker B motif (E274 and E678 at the NBD1 and NBD2) are replaced by alanine. This ClpB variant binds but not hydrolyses ATP^34,35^. The conformation of the ATP-state of the DnaK inactive mutants differs, as DnaK_K70A_ does not undergo the structural change that causes substrate dissociation and the modification of its intrinsic fluorescence properties, in contrast to DnaK_T199A_ that resembles the wt protein^34^. None of these DnaK mutants could activate the bichaperone mixture in the presence of protein aggregates (Fig. 3B and Supplementary Table S3), because they could not be recruited to the aggregate surface by DnaJ (Fig. 3C and Supplementary Fig. S3). Moreover, the low stimulation observed for wt DnaK in solution was neither observed for these DnaK mutants. Similarly, activation of the bichaperone mixture was absent when the inactive ClpB_TT_ variant and wt DnaK were used (Fig. 3B). These results suggest that activation of the DnaK-ClpB complex requires the conformational cycling, driven by ATP hydrolysis, of both chaperones.

**Figure 4:**
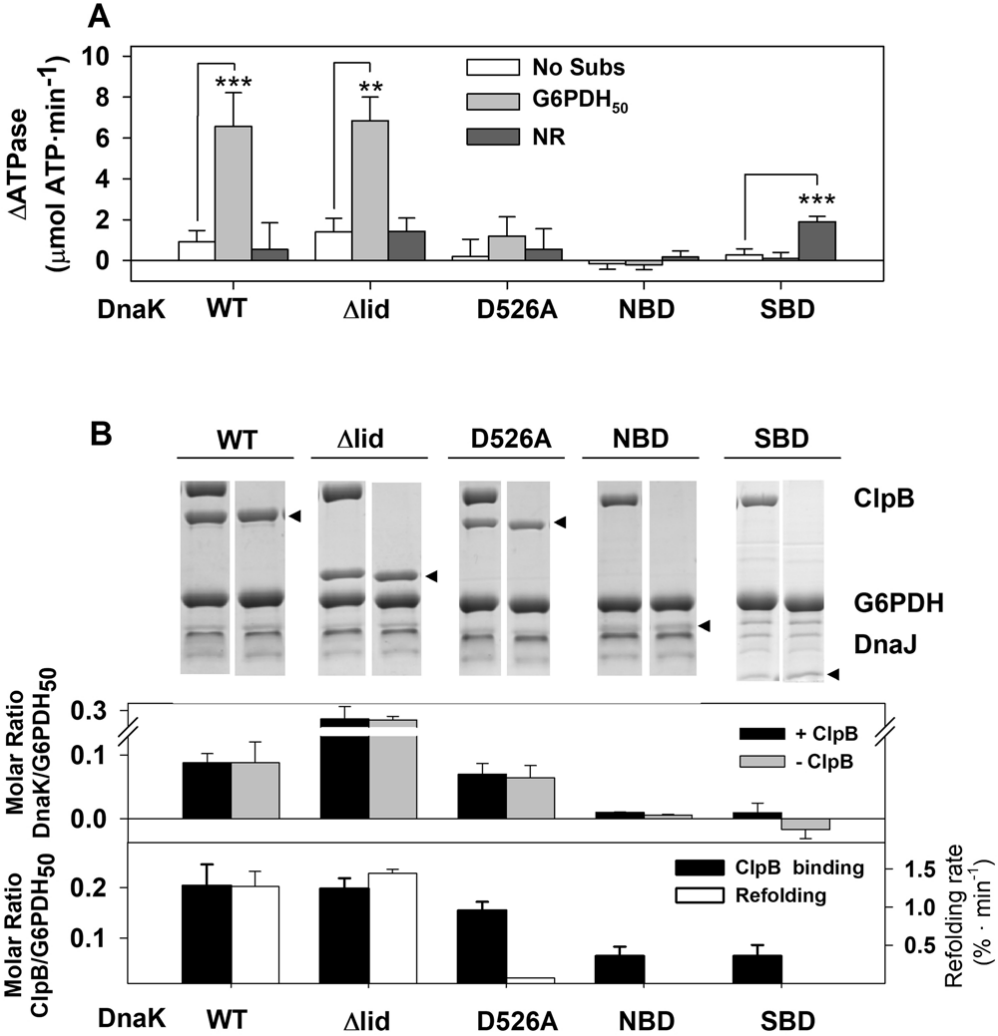
Structural regions of DnaK involved in the activation of the bichaperone complex. (**A**) Activation of the bichaperone complex by wt DnaK, DnaK_1–507_ (Δlid), DnaK_D526A_, DnaK_1–385_ (NBD) and DnaK_386–638_ (SBD + linker) in the absence (white bars), and presence of 1.2 µM G6PDH_50_ aggregates (grey bars) or NR peptide (dark grey bars). (**B**) Binding of wt and DnaK variants to protein aggregates in the presence or absence of ClpB. Other details as in Fig. 3. Pellets containing aggregate-bound chaperones were analyzed by 7.5% SDS-PAGE (upper panel) to estimate the amount of aggregate-bound DnaK (middle panel) or ClpB (lower panel; black bars), using known concentrations of the proteins as standards. The arrows highlight the position of the bands corresponding to the different DnaK variants. Reactivation rate of 1.2 µM G6PDH_50_ aggregates by bichaperone mixtures containing wt DnaK, DnaK_D526A_, DnaK_1–507_ (Δlid), DnaK_1–385_ (NBD) and DnaK_386–638_ (SBD + linker) (lower panel; white bars). Data are presented as mean ± s.d. of at least three independent experiments (**P < 0.01; ***P < 0.001).

Next, to characterize the structural elements of DnaK involved in the activation of the bichaperone complex, we measured the effect that deletion of the lid (DnaK_1–507_; Δlid), and the complete SBD (DnaK_1–385_; NBD) or NBD (DnaK_386–638_; SBD + linker) had on complex stimulation in the absence and presence of different client proteins (Fig. 4 and Supplementary Table S3). Removal of the lid had no effect on the activation of the bichaperone complex in the presence of aggregates (Fig. 4A) and facilitated binding of the chaperone to the aggregate surface where it recruited ClpB similarly to wt DnaK (Fig. 4B and Supplementary Fig. S3). The isolated NBD failed to form an activated complex with ClpB in all the conditions tested (Fig. 4A). The behaviour of the variant containing the SBD and the linker was somehow surprising. Whereas it was unable to generate a stimulated complex with ClpB in the presence of aggregates and in the absence of substrates, it could do so upon addition of a saturating concentration of the NR peptide (Fig. 4A). This unexpected result could be due to the presence of the hydrophobic linker of DnaK in our construct. The linker has been shown to mediate intermolecular DnaK associations^36,37^. NR binding to the SBD might facilitate exposure of the linker that could interact with and slightly activate ClpB, as found with NR and FAPPY (Supplementary Fig. S1 and Table S1). The lack of stimulation observed for the isolated domains in the presence of aggregates is expected as they are unable to interact with DnaJ^38^ and are therefore not transferred to the aggregate surface where the activating interaction with ClpB takes place (Fig. 4C and Supplementary Fig. S3). Furthermore, as we find here, it was also previously shown that the lid subdomain is not essential for the interaction with DnaJ^38^. Despite the fact that the lid is disposable to activate the bichaperone system in the presence of aggregates, the essential role of the proper allosteric interplay between both subdomains of the DnaK SBD is suggested by results obtained with the single point mutant DnaK_D526A_. The highly conserved D526 is part of a hinge responsible for the opening and closing of the binding site through conformational movements of the lid. The D526A mutation alters the conformation and dynamics of the lid in the ADP-bound, high affinity state for substrates, modifying interdomain interactions responsible for the stability of both protein domains^39^. Here we found that DnaK_D526A_ was unable to render a stimulated bichaperone complex (Fig. 4A), although it recruited ClpB at the aggregate surface (Fig. 4B and Supplementary Fig. S3). The disruption of the interactions between the lid and the β-SBD could likely affect the correct position of this subdomain in the aggregate-bound conformation, hampering the stimulation of the bichaperone complex. The ability of all the DnaK variants used above to activate the bichaperone complex was reflected in the rate of disaggregation, thus linking ATPase activation and disaggregation activity: wt- and Δlid-DnaK reactivated G6PDH_50_ aggregates whereas DnaK_D526A_ and the isolated NBD and SBD domains were inactive (Fig. 4B). These results suggest that proper allosteric displacement of the lid regulates the accessibility of the β-SBD,the structural element responsible for the productive activation of the aggregate-bound bichaperone complex.

### Activation of the bichaperone complex requires an active NBD2 and the integrity of the M-domain of ClpB

**Figure 5:**
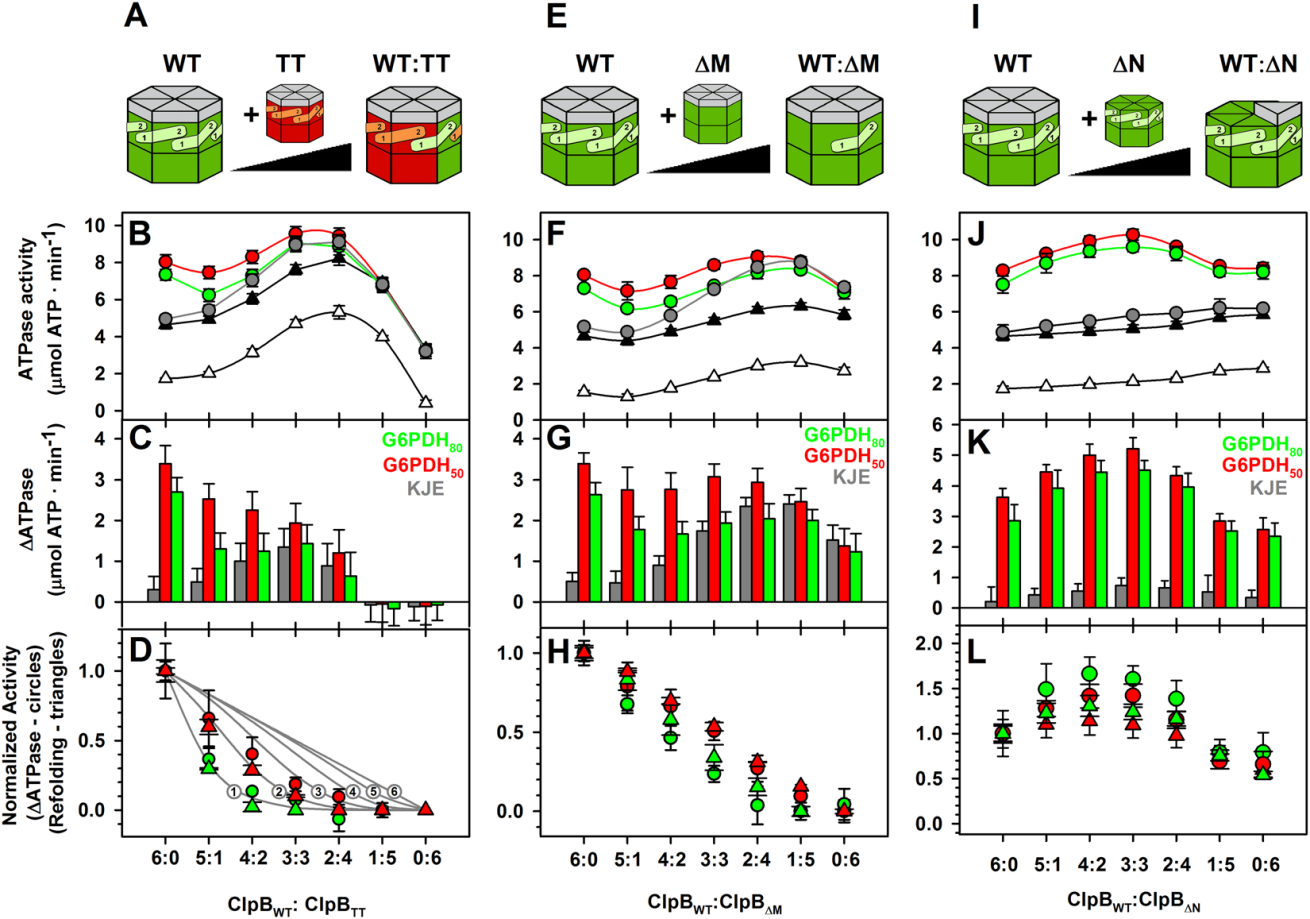
The ability of ClpB to form an activated complex with DnaK at the aggregate surface or in solution relies on intramonomeric communication of active protomers. (**A**,**E** and **I**) ClpB heterohexamer formation. Mixing of wt ClpB with increasing amounts of ClpB_E279A/E678A_ (ClpB_TT_) (**A**), ClpBΔ_(410–520)_ (ΔM-ClpB) (**E**) or ClpB_143–857_ (ΔN-ClpB) generates ClpB hybrids containing both types of subunits. (**B**,**F** and **J**) ATPase activity of ClpB hybrids composed of wt and ClpB_E279A/E678A_ (**B**), ClpBΔ_(410–520)_ (**F**), or ClpB_143–857_ (**J**) subunits, measured in the absence (empty triangles) and presence of the DnaK system alone (grey circles) or complexed with G6PDH_80_ (green symbols) or G6PDH_50_ (red symbols) aggregates. The expected ATPase activity values were obtained by adding the activities of each ClpB hexamer and the DnaK system, measured separately (black triangles). (**C**,**G** and **K**) Increase in the ATPase activity of mixtures of different ClpB species and the DnaK system in the absence (grey bars) or presence of G6PDH_80_ (green bars) or G6PDH_50_ (red bars). Stimulation was estimated by subtracting the expected ATPase activity (black triangles) from the experimental activity of the bichaperone complex in the absence of substrates (grey symbols), or in the presence of G6PDH_80_ (green symbols) or G6PDH_50_ (red symbols). (**D**,**H** and **L**) Normalized ATPase activity increase (circles) in the absence and presence of protein aggregates and normalized refolding rate (triangles) of ClpB_wt_:ClpB_TT_ (D), ClpB_wt_:ClpB_ΔM_ (**H**) and ClpB_wt_:ClpB_ΔN_ (**L**) hybrids in the presence of G6PDH_80_ (green symbols) or G6PDH_50_ (red symbols). The data for ClpB_wt_:ClpB_TT_ hybrids are compared with a binomial model assuming that hybrids are not stimulated or unable to reactivate aggregates when they contain one or more, two or more, three or more, four or more, five or more and six mutant subunits (grey lines labelled with the minimum number of mutant subunits that hamper complex stimulation or aggregate reactivation). Both ATPase activity and refolding rate were measured in the same experimental conditions: 2 µM DnaK, 0.7 µM DnaJ, 0.35 µM GrpE, 2 µM ClpB incubated in the absence or presence of 0.4 µM G6PDH aggregates. Data are presented as mean ± s.d. of at least three independent experiments.

**Figure 6:**
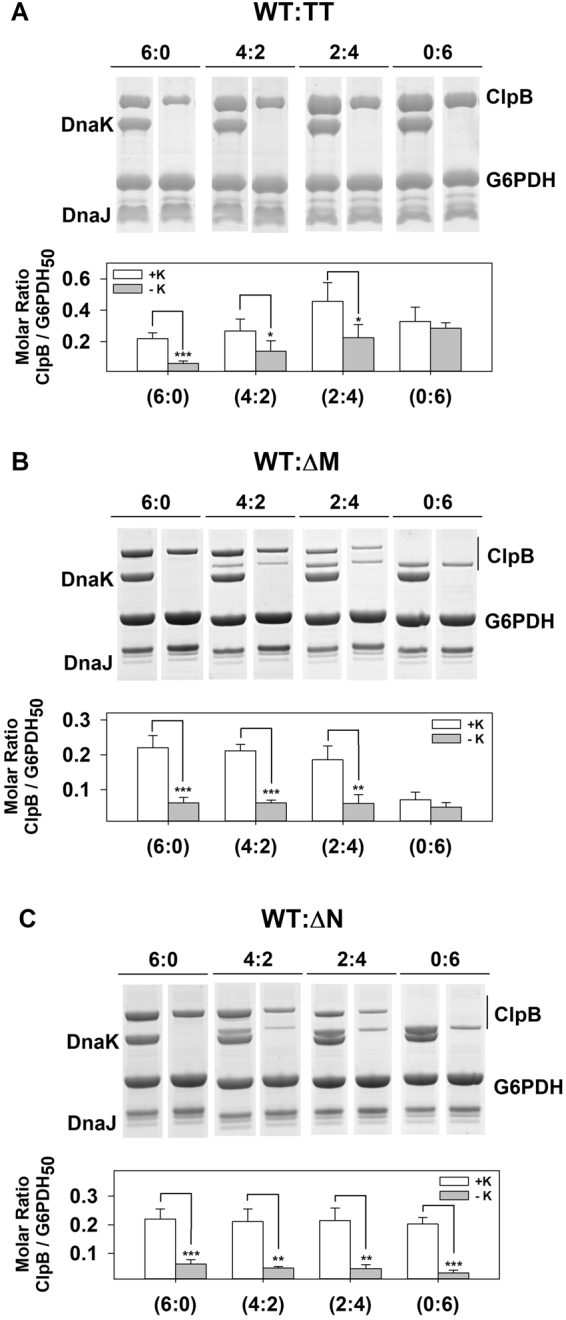
Two wt ClpB subunits ensure binding of ClpB hybrids to aggregates in a DnaK-dependent manner. Binding of ClpB heterohexamers made of wt and (**A**) ClpB_TT_, (**B**) ClpB_ΔM_, or (**C**) ClpB_ΔN_ subunits to protein aggregates. G6PDH_50_ aggregates were diluted to 1 μM in refolding buffer containing 3 mM ATP, DnaK (3.5 μM), DnaJ (0.7 μM), and 5 µM of the corresponding ClpB homo or heterohexamer. After an incubation of 10 min, samples were centrifuged to obtain the pellets containing aggregate-bound chaperones, which were analyzed by 4–12% Bis-Tris NuPAGE (upper panels) to estimate the amount of each ClpB homo and heterohexamer bound to the aggregate, using known amounts of each protein as standard (bottom panels). Data are shown as mean ± s.d. of three independent experiments (*P < 0.05; **P < 0.01; ***P < 0.001).

We next investigated the structural elements of ClpB that were implicated in the activation of the bichaperone complex, taking into account that the active species of the disaggregase is the hexamer. First, the role of the nucleotide binding domains (NBDs) was studied using the ClpB_TT_ mutant, able to bind but not hydrolyze ATP. Due to the inability of this mutant to activate the bichaperone complex, we used ClpB heterohexamers that contained different proportions of wt and ClpB_TT_ (Fig. 5A). Insertion of ClpB_TT_ subunits into the wt hexamer progressively increased the ATPase activity of the heterohexamers, indicating that the remaining wt monomers are stimulated by disruption of the intersubunit communication (Fig. 4B), in agreement with previous data^40,41^. In the absence of aggregates, activation of mixtures of DnaK and hybrid hexamers containing two or more mutant subunits was significantly higher than that observed for the wt ClpB hexamer (Fig. 5B and C). The opposite behaviour is observed in the presence of G6PDH aggregates, as incorporation of inactive subunits into the wt hexamer progressively abolished stimulation of the DnaK-ClpB complex (Fig. 5B and C). This could be due to a defective interaction of the ClpB hybrids with aggregates or aggregate-bound DnaK. However, they interacted with aggregates more efficiently than wt ClpB, and DnaK enhanced binding of these heterohexamers (Fig. 6A and Supplementary Fig. S4). In contrast, binding of ClpB_TT_ homohexamers did not depend on DnaK and in its absence interacted with protein aggregates four times better than wt ClpB (Fig. 6A and Supplementary Fig. S4). Therefore, the lack of activation observed for ClpB_wt_:ClpB_TT_ (2:4) hybrids is not due to a defective interaction with the aggregates, and points out that the subunit composition of the heterohexamer regulates stimulation of the bichaperone complex at the aggregate surface. These data indicate that an increase in the content of inactive subunits within the ClpB hexamer induces the loss of the activation of the bichaperone complex in the presence of aggregates, whereas it may favor the unproductive interaction with free DnaK. The activation of the bichaperone complex in the presence of protein aggregates can be simulated by a binomial model considering that it is abolished by the incorporation of 2–3 inactive subunits for aggregates formed at 50 °C and 1 for those obtained at 80 °C (Fig. 5D^42^;). The same hexamer composition dependence is noticed for the reactivation activity (Fig. 5D), suggesting that the energy derived from the stimulation of the bichaperone complex is used in substrate solubilization. The difference between the two types of aggregates might be due to the stronger mechanical demands required to solubilize G6PDH_80_ aggregates, which can only be provided by ClpB hexamers made of wt protomers.

Similar experiments were carried out with ClpB hybrids composed of wt and truncated ClpB subunits that lack the M (ΔM-ClpB) or N terminal (ΔN-ClpB) domains (Fig. 5E and I). We observed formation of hybrid hexamers with ΔM-ClpB despite the reported different oligomerization constants^12,43^, as insertion of truncated protomers resulted in the increase of the basal ATPase activity that deviates from linearity (Fig. 5F). This increment is most likely due to the disruption of the inhibitory signal coming from M domains of adjacent subunits^10,12^. In the absence of aggregated substrates, the activation of the DnaK/ΔM-ClpB complex was stronger than that observed with wt ClpB (Fig. 5F,G), suggesting that DnaK could also interact with a ClpB region different from the M domain, in agreement with the proposal that some residues of the N terminal domain form part of the complex interface^11^. The activation of the DnaK/hybrid complexes in solution (grey bars) increased with the relative amount of mutant subunits, indicating that the interaction is favored when neighboring subunits lack the M domain (Fig. 5F,G). In stark contrast, upon addition of protein aggregates, incorporation of ΔM-ClpB subunits into wt hexamers progressively diminished the stimulation of the bichaperone complex and its remodeling activity (Fig. 5F–H, red and green bars). This is not due to a defective interaction of the hybrids with aggregate-bound DnaK, as heterohexamers containing two or more wt subunits interacted similarly to wt ClpB, the DnaK-dependence for binding being lost only for the ΔM-ClpB homohexamer (Figs 6B and S4). Therefore, activation of the DnaK-ClpB complex at the aggregate surface and its subsequent remodeling activity depends, as aforementioned for the ClpB_wt_/ClpB_TT_ hybrids, on subunit composition of the disaggregase hexamer. These results suggest that the M domain is required to productively interact with aggregate-bound DnaK, preventing futile ATP consumption through the interaction with free DnaK. Deletion of the M-domain also abrogated the differences observed in the activation of the bichaperone complex in the absence and presence of protein aggregates or the NR peptide observed for wt ClpB (Fig. 2B), because the truncated disaggregase loses its capability to interact with aggregate-bound DnaK, as it does the ΔN/ΔM-ClpB variant (Fig. 2B and Supplementary Fig. S5). The lower activation found for the double deletion mutant, as compared with ΔM-ClpB, also suggests that the N domain could be a secondary interaction region for DnaK.

The basal ATPase activity of wt/ΔN-ClpB hybrids increased proportionally to the mutant protomer fraction^44^, even in the presence of the DnaK system (Fig. 5J). In contrast to what was found for the previous hybrids, mixing of wt/ΔN ClpB heterohexamers and DnaK in solution did not result in a significant activation^44^. In the presence of aggregates, wt/ΔN ClpB heterohexamers-DnaK complexes were stimulated more efficiently than those containing homohexamers. The stronger stimulation occurred at intermediate wt/ΔN ratios (Fig. 5K), explaining the higher disaggregation activity of these complexes (Fig. 5J–L). Hybrids and ΔN-ClpB homohexamers interacted with aggregate-bound DnaK similarly to wt ClpB (Fig. 6C and Supplementary Fig. S4). Thus, deletion of the N domain of ClpB does not hamper its ability to distinguish free and aggregate-bound DnaK. It is important to note that protein aggregates did not stimulate the different ClpB species in the absence of the DnaK system, or the DnaK system in the absence of ClpB (Supplementary Figs S1, S6 and Table S1).

**Figure 7:**
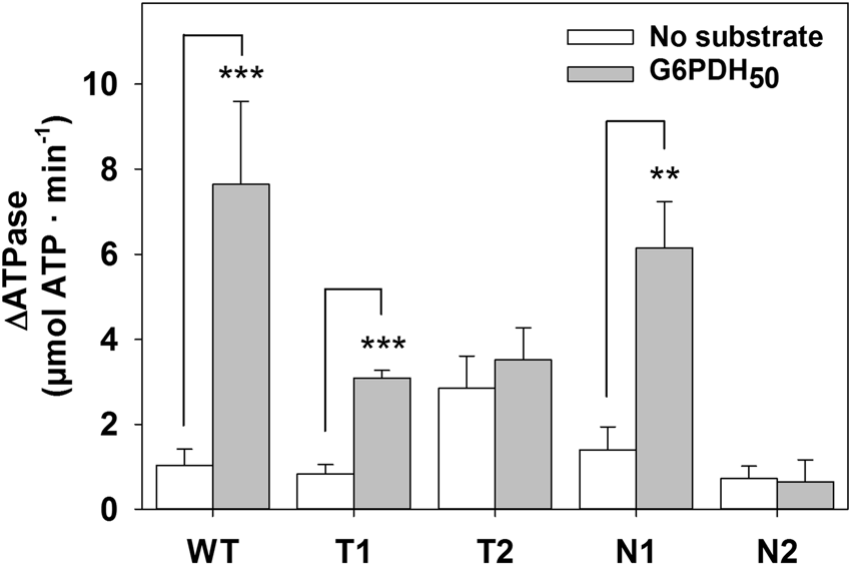
An active NBD2 is required for the stimulation of the aggregate-bound DnaK-ClpB complex. Activation of bichaperone mixtures formed by the DnaK system (3.5 μM DnaK, 0.7 μM DnaJ and 0.35μM GrpE) and 2 μM of wt or the corresponding ClpB variant. ClpB mutants contained only one of the two NBDs inactivated by the null (N1 or N2) or trap (T1 or T2) mutations described in Methods. Experiments were performed in buffer containing an ATPase regenerating system (2 mM ATP, 6 mM PEP and 20 ng/μl pyruvate kinase) at 25 °C in the absence or presence of 1.2 μM G6PDH_50_. The ΔATPase values were calculated as in Fig. 1. Data are shown as mean ± s.d. of three independent experiments (**P < 0.01; ***P < 0.001).

Next, we investigated whether ATP hydrolysis at each of the two NBD tiers had similar effect on the activation of the aggregate-bound bichaperone complex. To this aim, the same type of experiments were carried out with ClpB variants in which only one of the two NBDs harbours null (N) or trap (T) mutations that prevent nucleotide binding or hydrolysis, respectively^35,45^ (Fig. 7 and Table S4). Substitutions K212A and K611A at the Walker A motif in NBD1 and NBD2, respectively, abolish nucleotide binding (null –N- mutation), and mutations E274A and E678A at the Walker B motif allow binding but prevent hydrolysis at the mutated NBD (trap –T- susbtitution). The data showed that when ATP binding or hydrolysis at the NBD2 is abolished (ClpB_T2_ and ClpB_N2_), the DnaK/ClpB complex was not activated (Fig. 7). Besides nucleotide hydrolysis at the NBD2, full activation of ClpB also required a conformationally flexible NBD1 that could only be achieved in the absence of nucleotide binding (ClpB_N1_). ATP binding to this domain (ClpB_T1_) could “freeze” its conformation and modify the conformational cycling of structural elements, such as the M domain, involved in ATPase stimulation, thus resulting in a reduced activation. Taken together these data point to the integrity of the M-domain ring and ATP hydrolysis at the NBD2 as the essential features responsible for the stimulation of the aggregate-bound bichaperone complex.

## Discussion

In this report, we study how the functional association of the bacterial representatives of these chaperone families, DnaK and ClpB, is regulated. Previous studies have shown that activation of the DnaK-ClpB complex occurs at the aggregate surface, where it provides the energy for substrate remodeling^10,29,30^. This work explores how client proteins modulate the activation of the bichaperone complex and the structural elements of both proteins involved in this process. Our data show that activation depends on the client protein. While in the absence of substrates or presence of small peptides or RCMLA, a permanently unfolded model substrate^46^, the stimulation is weak, addition of larger partially or natively folded proteins, as α-casein and RepE, or protein aggregates induces a stronger activation. It is worth mentioning that the activation observed in the presence of protein aggregates arises from the small population of aggregate-bound DnaK-ClpB complexes^4,47^, suggesting that those engaged in ternary complexes are strongly activated. This interpretation is supported by the activation enhancement observed at increasing aggregate concentrations (compare Figs 1C and 3A), conditions that shift the binding equilibrium of DnaK, and hence ClpB, to their aggregate-bound states. The finding that the apparent affinity of ClpB for aggregate-bound DnaK is higher than for DnaK/peptide complexes or free DnaK, further proves that the disaggregase can discriminate DnaK complexed with protein aggregates that require its activation for remodeling. According to the estimated affinity, only aggregate-bound DnaK could efficiently recruit ClpB, forming an activated complex, at physiological disaggregase concentrations (9–20 μM)^48^.

Several protein-protein interactions are most likely behind activation of the bichaperone complex. The interaction of DnaK and ClpB with substrates induces structural rearrangements in both the chaperones and the client proteins. Our data suggest that these conformational changes are associated to the stimulation of the ATPase activity of the bichaperone complex. Aggregates and peptides might induce different conformational rearrangements in DnaK and only those brought about by aggregates favour formation of the activated complex. The structural properties of the substrates could select some configurations of the conformational space of DnaK and, possibly, ClpB. Those selected by aggregates and folded proteins, would be prone to interact, yielding an activated and productive complex.

Based on our results with DnaK-ClpB mixtures in which only one of the components is active, we cannot assign the observed stimulation of the ATPase activity to one chaperone as inactivation of either protein abolishes formation of an activated bichaperone complex. A recent study using similar inactive DnaK mutants fused to the N-terminus of ClpB from *Thermus thermophilus* has shown that *T*DnaK_K69A_ activates *T*ClpB, whereas TDnaK_K195A_ fails to stimulate the disaggregase. It was concluded that activation could mainly be attributed to *T*ClpB^49^. This apparent discrepancy might come from the different protein sources (*T*. *thermophilus* vs. *E*. *coli*) and/or the use of fusion proteins in this study. Formation of a bichaperone complex at the aggregate surface is not expected with the inactive DnaK mutants, as they do not interact with aggregate-bound DnaJ and therefore are not transferred to the aggregate surface. Similarly, the inactive ClpB_TT_ variant cannot stimulate DnaK in the presence of aggregates, although it binds better than wt ClpB to aggregates, but in a DnaK-independent manner. Therefore, DnaK-ClpB collaboration requires the coupled ATPase-dependent remodeling activities of both chaperones. A similar behavior was found for the DnaK-Hsp90 complex from *E*. *coli*, which was unable to remodel client proteins upon inactivation of any of the two chaperones^50^. The good correlation found between the DnaK-dependent stimulation of the ClpB subunit exchange process and the ATPase activity of the chaperone complex in the presence of *α*-casein, suggests that DnaK would activate the disaggregase^29^.

The main differences between the substrate proteins used in this study are their size and conformational properties. It has been shown that interaction with distinct substrates unleashes the conformational plasticity of DnaK SBD and other Hsp70 homologues, suggesting that they might adapt similarly to their wide variety of substrates^25,51,52^. The SBD of DnaK is built by two subdomains: a β-sandwich (β-SBD) that contains a hydrophobic binding pocket surrounded by long loops that protrude from the β strands, and an α-helical subdomain (α-SBD) that can fold over the β-sandwich and interacts with the loops, acting as a lid that controls the accessibility of the binding pocket^53,54^. FRET and EPR studies have shown that the degree of closure of the lid over the β-SBD depends on the client: when DnaK interacts with a peptide substrate in an extended conformation, the SBD predominantly adopts a lid-closed conformation, whereas the lid remains open or in an intermediate configuration when it binds to bulky, natively folded or molten globule like proteins, and most likely to large aggregates^25,27^. Native, bulky substrates or aggregates would select a lid-open, SBD conformation, which could facilitate exposure of the residues at the β-subdomain of the SBD responsible for the activation. This interpretation implies that the structural information required for activation of the DnaK-ClpB complex is located at the β-subdomain of the DnaK SBD, and that displacement of the lid allows ClpB to access this DnaK region. The findings that deletion of the lid subdomain does not abrogate the ability of the disaggregase to distinguish aggregate- and peptide-bound DnaK and that the NBD cannot activate the bichaperone complex, further supports that residues at the β-SBD are responsible for complex activation. However, this structural region has to be linked to the NBD, as the SBD alone is unable to activate the complex in the presence of aggregates. This dependence would be expected if the DnaK conformation involved in formation of the activated bichaperone complex is generated during the protein ATPase cycle, as it is that of ClpB. The ability of Δlid-DnaK to collaborate with ClpB in aggregate reactivation is surprising in light of previous studies showing the requirement of this structural region to refold chemically denatured luciferase in the absence of ClpB^53,55^. The nature of this discrepancy is yet unclear but it might be related to the different substrates used in these studies and/or the participation of ClpB in the reactivation process.

Results obtained with the DnaK_D526A_ variant, which displays a defective lid dynamics^39^, also indicate that although the lid is not directly involved in the activation, its correct allosteric displacement could allow exposure of the residues at the β-SBD involved in stimulation of the bichaperone complex. Nevertheless, lid detachment is not sufficient for the activation to occur, as the Δlid-DnaK/ClpB complex is not stimulated in the absence of substrates or presence of RCMLA and peptides. Therefore, a combination of lid detachment and conformation of the substrate-bound β-SBD could regulate activation of the bichaperone complex. This argument implies that the β-SBD is flexible enough to adopt different conformations upon binding distinct protein substrates. Several findings have put forward the conformational flexibility of the β-SBD: i) the fast hydrogen-deuterium exchange of the substrate-binding loops in the presence of ATP^56,57^; ii) the heterogeneity of loop L_3,4_ and β3 strand in the three-dimensional solution structure of the SBD obtained by NMR^58^; and iii) computational studies showing changes within the β-SBD around three hinge regions that can be considered as intrinsic motions^28,59^. Based on these findings, it has been postulated that the β-SBD samples (at least) two alternative conformations with different organization of the β strands and the hydrophobic pocket, and that the equilibrium between these alternative states could be regulated by the interaction with other structural regions of the protein (NBD, linker and lid) or with substrates^28^. Our data suggest that the size and conformational properties of the substrates could help to stabilize some of these conformations. Disruption of the interactions between the lid and the β-SBD upon lid displacement would increase the flexibility of the β-SBD and its ability to adopt different conformations in complex with distinct substrates. The interplay between the two subdomains of the DnaK SBD to activate ClpB could have an important functional consequence, as it would ensure ClpB activation only when substrate remodeling is needed, avoiding an unregulated unfoldase cellular activity that can be toxic for the cell^10,13,15^.

The ability of ClpB to distinguish different substrate-bound DnaK conformations depends on the integrity of the M-domain ring and on the activity of its NBD2 tier. They could be related, as the relative position of the M domain might be influenced by ATP hydrolysis at the NBD2. Indeed, the recent cryoEM structure of a substrate-acceptor state of ClpB places the NBD1, where the M domain is inserted, of one subunit on top of the NBD2 of the next one, thus providing a plausible pathway for inter-subunit and inter-tier communication^8^. This structure also reveals that the NBD2 undergoes pronounced conformational changes upon substrate binding, suggesting that it is the main disaggregase motor. In this context our data also show that its activity is essential for the activation of the bichaperone complex at the aggregate surface. When the physical integrity of the M-domain ring is disrupted, the disaggregase can be unproductively stimulated by free DnaK. This finding provides a new function to this unique structural element, namely to distinguish the different substrate-bound DnaK conformations. These conformations might display different affinities for its various partners, as ClpB, which would direct the chaperone to substrate remodeling, or GrpE, which will engage DnaK in protein refolding.

## Methods

### Protein expression and purification

Wild-type ClpB and its mutants were expressed in *Escherichia coli* BB4561 strain and purified as previously described^60^. Mutation of the conserved lysine residues at the Walker A motif (K212 and K611 in NBD1 and NBD2, respectively) by alanine impairs nucleotide binding at the mutated NBD (null –N- mutation), whereas substitution of the conserved glutamic residues at the Walker B motif (E274 and E678 at the NBD1 and NBD2) by alanine renders NBDs that bind but not hydrolyse ATP (trap –T- susbtitution^35^). Full length (wt, ClpB_TT_ -ClpB_E279A/E678A_-, ClpB_T1_ -ClpB_E279A_-, ClpB_T2_ -ClpB_E678A_-, ClpB_N1_ -ClpB_K212A_- and ClpB_N2_ -ClpB_K611A_-) and deletion mutants (ΔN-ClpB -ClpB_Δ(1–142)_, ΔM-ClpB -ClpB_Δ(410–520),_ and ΔN/ΔM-ClpB –ClpB_Δ(1–142;410–520)_) of ClpB were obtained as previously reported^12,41,61^. Wt DnaK, DnaK_T199A_, DnaK_D526A_, Δlid (DnaK_1–507_), NBD (DnaK_1–385_), DnaJ and GrpE were obtained as reported^39,62^. DnaK SBD containing the linker (DnaK_386–638_) was cloned as a SUMO fusion construct and purified by successive Ni-NTA chromatography steps, after proteolysis with Ulp1. DnaK_K70A_ was obtained by QuickChange and purified as the wt protein. RepE was expressed and purified as previously described^63^. Protein concentration was determined by the colorimetric Bradford assay (Bio-Rad Laboratories). Glucose-6-phosphate dehydrogenase (G6PDH), and α-casein were purchased from Sigma, and Lactate dehydrogenase and pyruvate kinase from Roche Applied Science. RCMLA was produced from α-lactalbumin (Sigma) as described^64^. All chaperone concentrations are given for their monomeric species. F-APPY (fluorescein-CALLQSRLLLSAPRRAAATAR) and peptide NR (NRLLLTG) were obtained from NeoMPS and Proteogenix, respectively.

### Protein aggregate reactivation

G6PDH (10 μM) was denatured at 50 (G6PDH_50_) or 80 °C (G6PDH_80_) for 30 min in 50 mM Tris-HCl (pH 7.5), 150 mM KCl, 20 mM MgCl_2_ and 10 mM DTT. Denatured and aggregated G6PDH was diluted to 0.4–1.6 μM in the same buffer containing 50 mM KCl. Reactivation was started after the addition of 2 mM ATP to the sample containing 0.7 μM DnaJ, 0.35 μM GrpE, 3.5 μM DnaK, 2 μM ClpB containing the indicated molar ratio of wt and mutant subunits, and an ATP-regeneration system (6 mM phosphoenolpyruvate and 20 ng/ml pyruvate kinase). G6PDH activity was measured at 30 °C as previously described^65^, in a Synergy microplate reader using 96-well plates. ClpB hybrids were prepared by mixing different amounts of ClpB_wt_ and the corresponding mutant, keeping the total ClpB concentration constant.

### ATPase activity

Steady-state ATPase activity was measured in 50 mM Tris-HCl (pH 7.5), 50 mM KCl, 20 mM MgCl_2_ at 25 °C, as previously described^62^ in the presence of an ATP-regenerating system^29^. Final ClpB and ATP concentrations were 2 μM and 2 mM, respectively. The activity of the same samples was also measured in the presence of 3.5 μM DnaK, 0.7 μM DnaJ, and 0.35 μM GrpE, to study the DnaK-induced activation of the disaggregase. Substrate-induced stimulation of the ATPase activity was determined in the presence of 0.4–1.6 μM G6PDH aggregates formed at 50 or 80 °C, 10 μM α-casein, 5 µM RepE, 10 µM RCMLA, 10 μM F-APPY, or 350 μM NR peptide (NRLLLTG). Reactions were followed by measuring the absorbance decay at 340 nm in a Synergy microplate reader using 96-well plates, at 25 °C. The concentration of all the substrates was saturating, except that of protein aggregates. The scattering of these samples at higher concentrations precluded the use of the coupled assay employed in these measurements.

### Dynamic Light Scattering (DLS)

DLS measurements were performed at 25 °C in 50 mM Tris-HCl (pH 7.5), 50 mM KCl, 20 mM MgCl_2_ using a Nano-S Zetasizer (Malvern Instruments, UK) and 173° backscatter detection. Protein concentration was 0.4 µM G6PDH aggregates, 10 μM α-casein, 5 µM RepE and 10 µM RCMLA. Data were analyzed with the Zetasizer family software considering the viscosity and refractive index of the buffer.

### Chaperone binding to protein aggregates

Association of chaperones with G6PDH aggregates was estimated by electrophoresis of the pellets obtained after centrifugation of the protein mixtures, as described^4^. G6PDH_50_ aggregates (1 μM) were incubated 10 min at 30 °C in refolding buffer with 1 μM DnaJ, 3.5 μM wt or the desired DnaK variant, and 5 μM of wt or mutant ClpB, in the presence of ATP regenerating system (3 mM ATP, 6 mM phosphoenolpyruvate and 20 ng/ml pyruvate kinase). GrpE was not added to avoid aggregate reactivation. Samples were centrifuged at 4 °C in a Beckman Optima ultracentrifuge at 122,000 × g for 40 min, to separate free and aggregate-bound chaperones. The resulting pellets and controls, containing known amounts of native proteins, were analyzed by SDS–PAGE (7.5%) or NuPAGE (4–12% Bis-Tris) (Novex, Life Technologies), as indicated. The amount of aggregate-bound chaperones, relative to that of the aggregated protein, was estimated by densitometry of the gel bands with a gel scanner G-800 and the Quantity One software (Bio-Rad). Each data point is the average of at least three independent experiments and was estimated by subtracting the amount of protein found in pellets of control experiments, containing native G6PDH.

### Modeling the activity of ClpB heterohexamers

To explore the conformational elements involved in DnaK-mediated activation of ClpB, we have used a previously described binomial distribution model^42^. This model predicts the probability of each oligomeric species to occur upon mixing wt and mutant subunits at a defined molar ratio, allowing the comparison with the experimental data.

### Statistical analysis

All measurements were performed at least 3 times and levels of significance were determined by a two-tailed Student’s t-test. A value of P < 0.05 was considered statistically significant (*P < 0.05; **P < 0.01; ***P < 0.001).

## Electronic supplementary material

Supplementary Information
