# Supplementary material for: Activation of the DnaK-ClpB Complex is Regulated by the Properties of the Bound Substrate
Source: Sci Rep. 2018 Apr 11;8:5796. doi: 10.1038/s41598-018-24140-5 (PMC5895705; doi:10.1038/s41598-018-24140-5)
Supplement: Supplementary file 1 — Supplementary Information [file 41598_2018_24140_MOESM1_ESM.pdf]

## SUPPLEMENTARY INFORMATION

# ACTIVATION OF THE DnaK-ClpB COMPLEX IS REGULATED BY THE PROPERTIES OF THE BOUND SUBSTRATE

Jose Angel Fernández-Higuero, Alejandra Aguado, Judit Perales-Calvo, Fernando Moro and Arturo Muga*

Biofisika Institute (CSIC, UPV/EHU) and Department of Biochemistry and Molecular Biology, Faculty of Science and Technology, University of the Basque Country (UPV/EHU), P.O. Box 644, 48080 Bilbao, Spain.

* Corresponding author: arturo.muga@ehu.eus

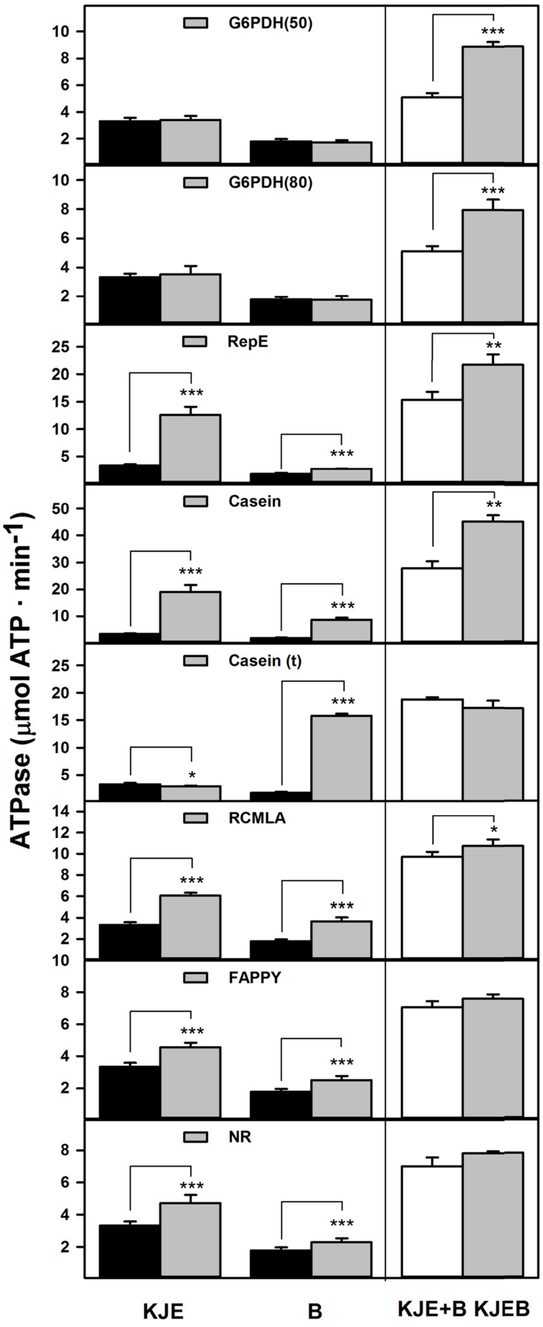


**Figure S1. Effect of different protein substrates on the ATPase activity of the DnaK system, ClpB and their mixture.** (Left panel) ATPase activity of the DnaK system (KJE) or ClpB in the absence (black bars) or presence (grey bars) of substrates. (Right panel) Expected (white bars) and experimental (grey bars) activity of the mixture of both chaperones under the same experimental conditions. 3.5 μM DnaK, 0.7μM DnaJ, 0.35 μM GrpE or/and 2 μM ClpB were incubated in the absence or presence of the following substrates: 0.4 μM $G6PDH_{50}$ or $G6PDH_{80}$ aggregates, 5 μM RepE, 10 μM Casein (full length or trypsinized –t-), 10 μM RCLMa, 10 μM FAPPY or 350 μM NR peptide. Data are shown as mean ± s.d. of n ≥ 3 independent experiments (**P*<0.05; ***P*<0.01; ****P*<0.001).

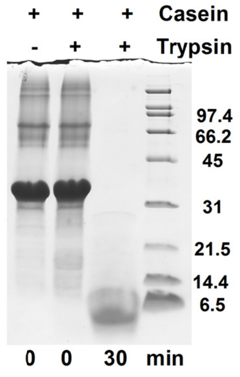


**Figure S2. Casein digestion with trypsin.** Casein (4.8 μg/μl) was incubated with trypsin at 500:1 ratio in 50 mM Tris-HCl, 5 mM $MgCl_2$, 50 mM KCl and pH 7.5 buffer at 30ºC. The reaction was stopped by the addition of 1 mM (final concentration) of PMSF. Samples were afterwards analysed by SDS-PAGE.

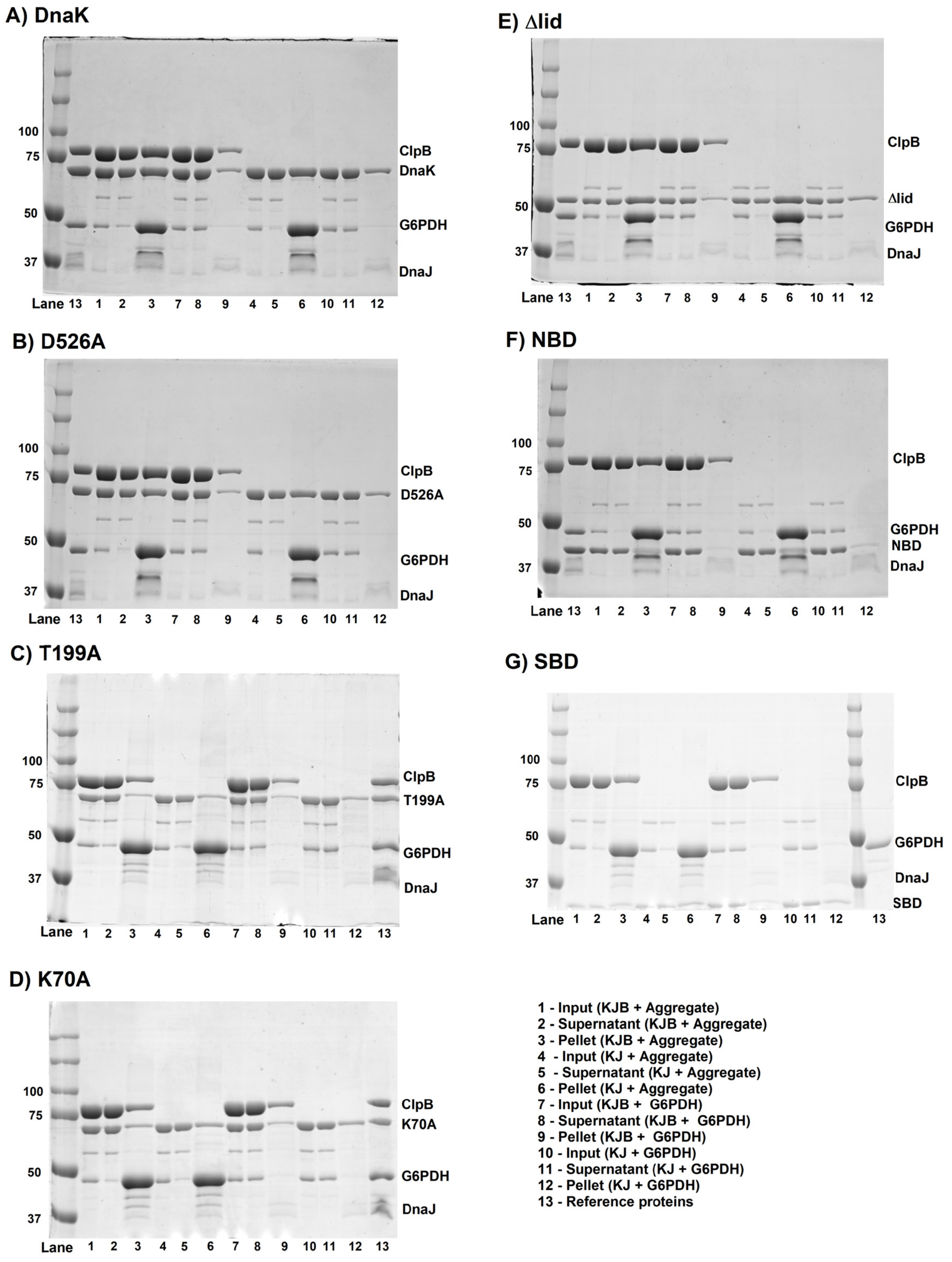


**Figure S3. Binding of DnaK variants to aggregates in the presence and absence of ClpB.** $G6PDH_{50}$ aggregates were diluted to 1 µM in refolding buffer containing 3 mM ATP, DnaK (3.5 µM), DnaJ (0.7 µM) without GrpE to slow down aggregate reactivation. When present, ClpB concentration was 5 µM. Samples were incubated 10 min and centrifuged to separate free and aggregate-bound chaperones. The inputs (4% of the initial sample), supernatants, mixtures of known protein concentration (reference proteins), and pellets obtained in the presence of $G6PDH_{50}$ (aggregate) and native substrate (G6PDH) were analyzed by 7.5% SDS-PAGE to estimate the amount of aggregate-bound DnaK shown in Fig. 3.

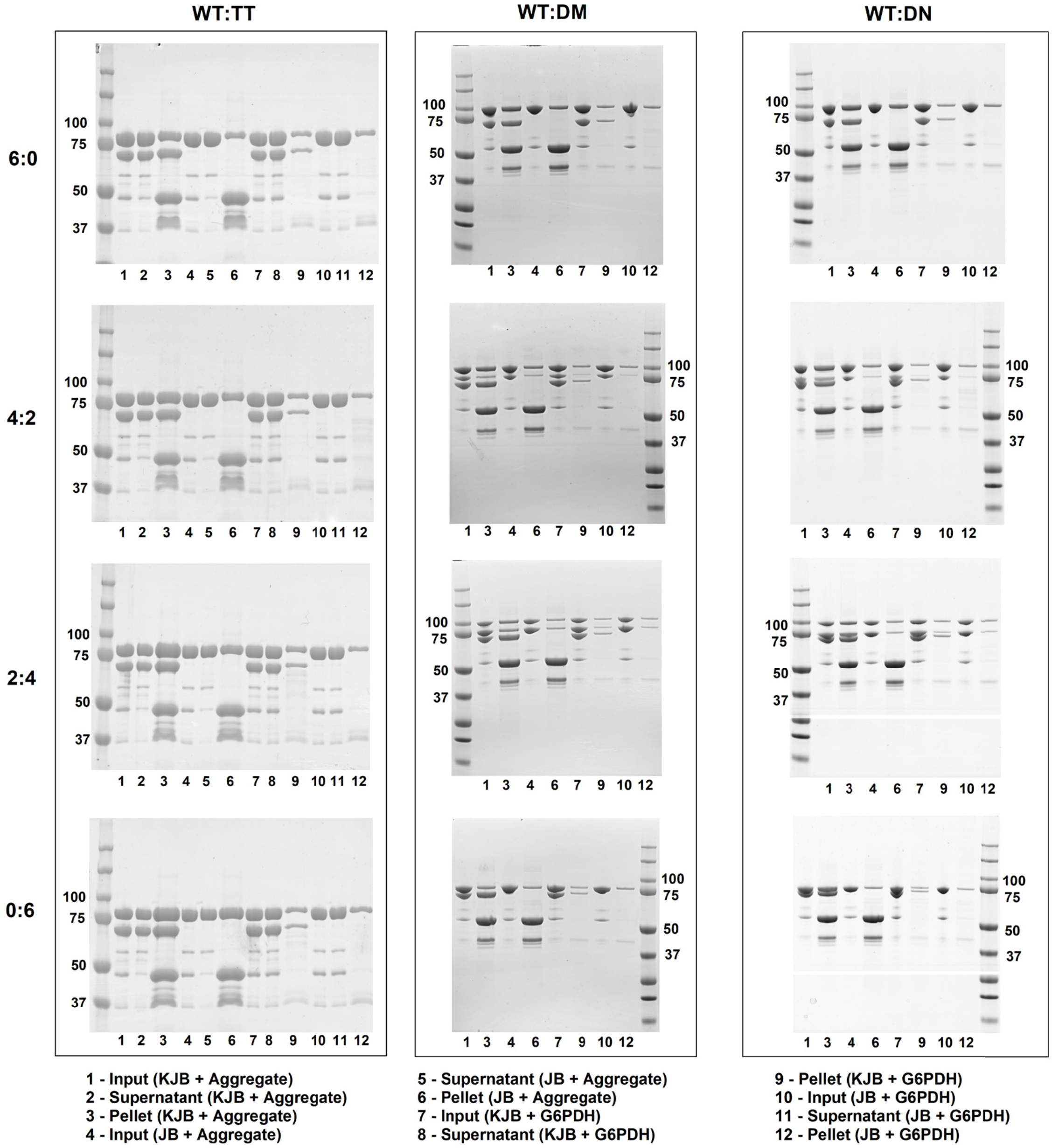


**Figure S4. Binding of ClpB homohexamers and heterohexamers containing different molar ratios of wt and ClpB$_{TT}$ (left panel), ClpB$_{\Delta M}$ (middle panel), or ClpB$_{\Delta N}$ (right panel) subunits.** G6PDH$_{50}$ aggregates were diluted to 1 μM in refolding buffer containing 3 mM ATP, DnaJ (0.7 μM) and 5 μM of the corresponding ClpB homo or heterohexamer, in the absence or presence of DnaK (3.5 μM). Samples were centrifuged after 10 min incubation to obtain the pellets containing aggregate-bound chaperones, which together with the inputs, containing known amounts of each protein (4% of the initial sample), and the supernatants were analyzed by 7.5% SDS-PAGE (WT:TT) or 4-12% Bis-Tris NuPAGE (WT:ΔM and WT:ΔN). The same experiments were performed with native G6PDH, and used as controls of protein sedimentation.

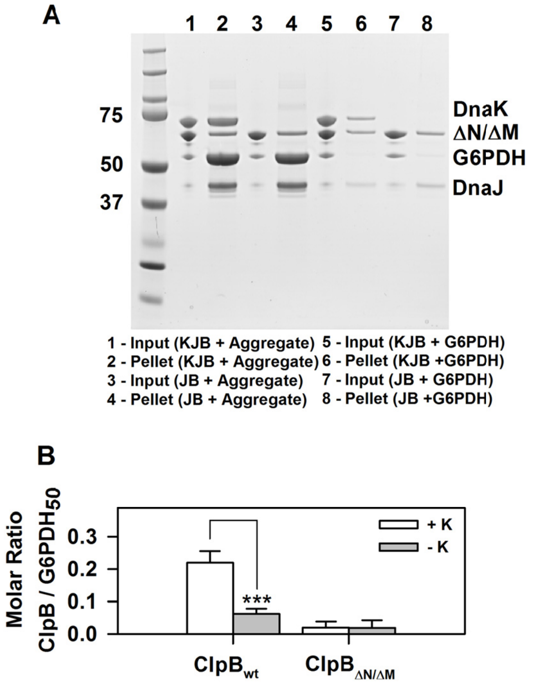


**Figure S5. The ΔN/ΔM-ClpB variant does not interact with aggregate-bound DnaK.** (A) Samples containing 1 µM $G6PDH_{50}$, 3 mM ATP, 0.7 µM DnaJ, 3.5 µM DnaK and 5 µM ΔN/ΔM-ClpB were incubated 10 min in refolding buffer and centrifuged to obtain the pellets containing aggregate-bound chaperones. Inputs and pellets of these samples and of those obtained under the same experimental conditions using native (G6PDH), instead of aggregated substrate, were analyzed by 4-12% Bis-Tris NuPAGE. (B) Estimation of the amount of ΔN/ΔM-ClpB bound to the aggregate in the presence (white bar) and absence (grey bar) of DnaK. Results for wt ClpB are also shown for the sake of comparison. Data are shown as mean ± s.d. of n ≥ 3 independent experiments (****P*<0.001).

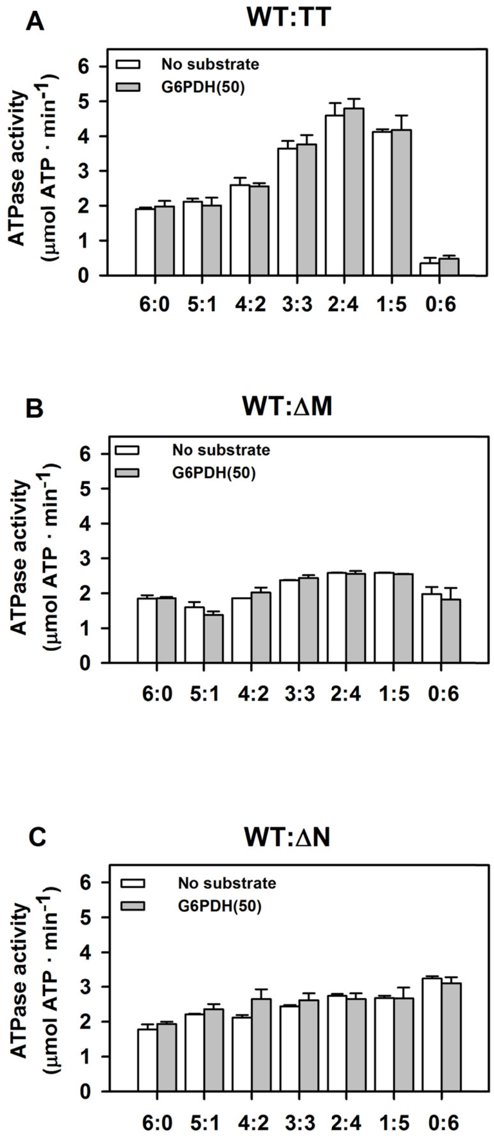


**Figure S6. Aggregates of G6PDH do not modify the ATPase activity of the different ClpB homohexamers and hybrids.** ATPase activity of the chaperone mixture containing 3.5 µM DnaK, 0.7µM DnaJ, 0.35 µM GrpE and 2 µM ClpB in the absence (black bars) and presence (grey bars) of 0.4 µM $G6PDH_{50}$. ClpB heterohexamers contain different molar ratios of wt ClpB and $ClpB_{TT}$ (A), $ClpB_{\Delta M}$ (B), or $ClpB_{\Delta N}$ (C) subunits. Experimental details as in Fig. 5. Data are shown as mean ± s.d. of n ≥ 3 independent experiment.

| | KJE | *P*-value No subs. vs. Subs | B | *P*-value No subs. vs. Subs. | (KJE+B) | KJEB | *P*-value (KJE+B) vs. KJEB | KJEB - (KJE+B) | *P*-value No subs vs. Subs |
|---|---|---|---|---|---|---|---|---|---|
| **No subs.** | 3.32 ± 0.26 | | 1.78 ± 0.19 | | 5.10 ± 0.32 | 5.93 ± 0.49 | ≤ 0.001 | 0.84 ± 0.58 | |
| **$G6PDH_{50}$** | 3.42 ± 0.30 | 0.604 | 1.70 ± 0.18 | 0.458 | 5.12 ± 0.35 | 8.86 ±0.37 | ≤ 0.001 | 3.75 ± 0.51 | ≤ 0.001 |
| **$G6PDH_{80}$** | 3.54 ± 0.56 | 0.960 | 1.76 ± 0.27 | 0.857 | 5.29 ± 0.62 | 7.94 ± 0.72 | ≤ 0.001 | 2.65 ± 0.95 | 0.001 |
| **RepE** | 12.61 ± 1.48 | ≤0.001 | 2.75± 0.04 | ≤0.001 | 15.35 ± 1.48 | 21.70 ± 1.92 | 0.002 | 6.34 ± 2.43 | ≤ 0.001 |
| **CAS** | 19.11 ± 2.53 | ≤0.001 | 8.72 ± 0.73 | ≤0.001 | 27.82 ± 2.63 | 45.17 ± 2.37 | 0.005 | 17.35 ± 3.54 | ≤ 0.001 |
| **CAS(t)** | 2.98 ± 0.13 | 0.028 | 15.79 ± 0.36 | ≤0.001 | 18.77 ± 0.38 | 17.59 ± 1.37 | 0.215 | -1.18 ± 1.42 | 0.007 |
| **RCMLA** | 6.06 ± 0.27 | ≤0.001 | 3.66 ± 0.38 | ≤0.001 | 9.72 ± 0.46 | 10.73 ± 0.61 | 0.038 | 1.02 ± 0.77 | 0.655 |
| **FAPPY** | 4.54 ± 0.29 | ≤0.001 | 2.51 ± 0.24 | ≤0.001 | 7.06 ± 0.37 | 7.59 ± 0.27 | 0.241 | 0.54 ± 0.46 | 0.527 |
| **NR** | 4.71 ± 0.51 | ≤0.001 | 2.29 ± 0.25 | ≤0.001 | 7.01 ± 0.56 | 7.83 ± 0.12 | 0.059 | 0.82 ± 0.58 | 0.973 |

**Table S1**. ATPase activity (μmol ATP·$min^{-1}$) values obtained for the isolated DnaK system (KJE) and ClpB (B), the arithmetic addition of these activities (KJE+B), and the experimental activity of mixtures of both chaperones (KJEB), in the absence and presence of different client proteins, as described in Fig. S1. Data are shown as mean ± s.d. of n ≥ 3 independent experiments. *P* values obtained from a two-tailed Student´s t-test are also shown.

| | ATPase activity (µmol ATP · min$^{-1}$) | | | | | |
|---|---|---|---|---|---|---|
| **ClpB** | **No substrate** | | **G6PDH$_{50}$** | | **NR** | |
| **WT (µM)** | **- KJE** | **+ KJE** | **- KJE** | **+ KJE** | **- KJE** | **+ KJE** |
| 0 | | 3.15 ± 0.23 | | 3.16 ± 0.11 | | 4.96 ± 0.17 |
| 1 | 0.69 ± 0.06 | 4.18 ± 0.54 | 0.70 ± 0.04 | 10.05 ± 0.63 | 0.93 ± 0.03 | 6.64 ± 0.16 |
| 2 | 1.00 ± 0.12 | 5.02 ± 0.62 | 0.96 ± 0.04 | 12.97 ± 0.84 | 1.74 ± 0.11 | 7.66 ± 0.04 |
| 4 | 1.63 ± 0.10 | 5.99 ± 0.69 | 1.54 ± 0.07 | 15.69 ± 0.48 | 3.72 ± 0.11 | 9.93 ± 0.13 |
| 6 | 2.27 ± 0.18 | 7.75 ± 0.43 | 2.18 ± 0.17 | 19.06 ± 1.65 | 5.46 ± 0.14 | 11.98 ± 0.07 |
| 8 | 2.94 ± 0.23 | 8.82 ± 0.51 | 2.78 ± 0.10 | 20.37 ± 0.35 | 7.25 ± 0.25 | 13.70 ± 0.10 |
| 10 | 3.66 ± 0.20 | 10.08 ± 0.48 | 3.65 ± 0.19 | 23.37 ± 0.79 | 9.47 ± 0.24 | 16.25 ± 0.19 |
| 15 | 5.88 ± 0.49 | 13.32 ± 0.27 | 5.39 ± 0.29 | 26.22 ± 0.33 | 12.50 ± 0.23 | 20.21 ± 0.20 |
| 20 | 8.20 ± 0.70 | 16.69 ± 0.43 | 7.72 ± 0.15 | 30.09 ± 0.43 | 16.90 ± 0.54 | 25.61 ± 0.60 |
| 25 | 11.43 ± 0.64 | 21.02 ± 1.44 | 10.75 ± 0.04 | 34.34 ± 0.06 | 21.49 ± 0.41 | 31.13 ± 0.48 |
| 30 | 14.85 ± 0.82 | 25.39 ± 0.61 | 13.02 ± 0.44 | 37.33 ± 0.68 | 27.06 ± 0.64 | 37.32 ± 0.98 |
| **ΔM (µM)** | **- KJE** | **+ KJE** | **- KJE** | **+ KJE** | **- KJE** | **+ KJE** |
| 0 | | 3.19 ± 0.33 | | 3.22 ± 0.24 | | 5.17 ± 0.18 |
| 1 | 0.63 ± 0.07 | 2.98 ± 0.34 | 0.57 ± 0.10 | 3.19 ± 0.21 | 1.90 ± 0.11 | 7.12 ± 0.23 |
| 2 | 1.41 ± 0.10 | 5.12 ± 0.43 | 1.36 ± 0.08 | 4.71 ± 0.48 | 5.90 ± 0.20 | 11.94 ± 0.17 |
| 4 | 3.00 ± 0.24 | 8.38 ± 0.41 | 2.55 ± 0.11 | 7.71 ± 0.49 | | |
| 6 | 4.82 ± 0.31 | 12.59 ± 0.78 | 4.14 ± 0.32 | 9.76 ± 0.76 | 20.33 ± 0.94 | 29.41 ± 1.53 |
| 8 | 7.00 ± 0.59 | 15.40 ± 0.76 | 6.28 ± 0.22 | 12.78 ± 0.49 | | |
| 10 | 9.76 ± 0.31 | 19.96 ± 0.77 | 8.16 ± 0.47 | 16.82 ± 0.76 | 29.33 ± 1.01 | 42.14 ± 1.74 |
| 15 | 16.25 ± 1.15 | 30.37 ± 2.29 | 14.31 ± 0.49 | 24.76 ± 0.73 | | |
| 20 | 22.77 ± 0.77 | 41.55 ± 2.10 | 21.43 ± 0.52 | 36.57 ± 0.87 | 53.87 ± 1.77 | 73.21 ± 3.20 |
| 30 | 38.42 ± 0.47 | 62.94 ± 1.95 | 34.76 ± 0.84 | 55.20 ± 1.65 | | |
| **ΔN/ΔM (µM)** | **- KJE** | **+ KJE** | **- KJE** | **+ KJE** | **- KJE** | **+ KJE** |
| 0 | | 3.37 ± 0.16 | | 3.34 ± 0.33 | | 5.28 ± 0.49 |
| 2 | 0.79 ± 0.11 | 4.09 ± 0.54 | 0.61 ± 0.07 | 3.78 ± 0.27 | 3.01 ± 0.58 | 8.11 ± 0.32 |
| 4 | 1.46 ± 0.07 | 4.76 ± 0.48 | 1.07 ± 0.08 | 4.34 ± 0.27 | 6.44 ± 1.69 | 12.76 ± 0.58 |
| 10 | 3.51 ± 0.38 | 7.99 ± 1.22 | 3.35 ± 0.22 | 7.67 ± 1.51 | 20.81 ± 0.27 | 28.08 ± 0.39 |
| 20 | 7.08 ± 0.27 | 12.89 ± 1.22 | 6.26 ± 0.30 | 12.39 ± 1.62 | 43.25 ± 1.26 | 52.34 ± 1.40 |
| 30 | 12.83 ± 0.49 | 20.25 ± 1.46 | 11.73 ± 0.58 | 19.59 ± 1.18 | 69.01 ± 2.68 | 78.30 ± 2.69 |

**Table S2**. ATPase activity of samples lacking the DnaK system or containing 3.5 µM DnaK, 0.7µM DnaJ, 0.35 µM GrpE and increasing concentrations of wt ClpB, ΔM-ClpB or ΔN/ΔM-ClpB. Experiments were carried out in the absence of client proteins and in the presence of 1.2 µM G6PDG$_{50}$ or 350 µM NR. Data are shown as mean ± s.d. of at least three independent experiments.

| | ATPase Activity (µmol ATP · min$^{-1}$) | | |
|---|---|---|---|
| | **No substrate** | **G50 (1.2uM)** | **NR** |
| **K** | 0.59 ± 0.06 | 0.75 ± 0.12 | |
| **B** | 1.96 ± 0.25 | 1.92 ± 0.16 | 2.34 ± 0.23 |
| **KJ** | 3.17 ± 0.15 | 2.65 ± 0.07 | |
| **KE** | 1.60 ± 0.05 | 1.11 ± 0.29 | |
| **KB** | 2.65 ± 0.15 | 2.82 ± 0.18 | |
| **KJE** | 3.12 ± 0.20 | 3.18 ± 0.70 | 5.48 ± 0.70 |
| **KJB** | 5.69 ± 0.24 | 10.20 ± 0.33 | |
| **KEB** | 3.74 ± 0.51 | 3.02 ± 0.17 | |
| **(D526A)JE** | 3.67 ± 0.74 | 3.31 ± 0.72 | 12.66 ± 0.71 |
| **(Δlid)JE** | 4.87 ± 0.28 | 5.47 ± 0.74 | 9.38 ± 0.35 |
| **(NBD)JE** | 0.42 ± 0.10 | 0.47 ± 0.02 | 0.44 ± 0.04 |
| **KJEB** | 5.98 ± 0.47 | 11.67 ± 1.49 | 8.37 ± 1.08 |
| **(T199A)JEB** | 2.05 ± 0.15 | 1.79 ± 0.21 | 2.89 ± 0.05 |
| **(K70A)JEB** | 2.27 ± 0.22 | 1.85 ± 0.23 | 2.86 ± 0.19 |
| **KJE(TT)** | 3.51 ± 0.63 | 2.98 ± 0.43 | |
| **(D526A)JEB** | 5.81 ± 0.35 | 6.42 ± 0.60 | 15.55 ± 0.67 |
| **(Δlid)JEB** | 8.22 ± 0.56 | 14.23 ± 0.88 | 13.15 ± 0.50 |
| **(NBD)JEB** | 2.23 ± 0.06 | 2.18 ± 0.17 | 2.95 ± 0.17 |
| **(SBD)JEB** | 2.24 ± 0.13 | 2.02 ± 0.25 | 4.23 ± 0.10 |

**Table S3.** ATPase activities of different combinations of the DnaK variants used in this study with the cochaperones DnaJ and GrpE, and with wt ClpB or its inactive $ClpB_{TT}$ mutant. The ATP hydrolysis rate was measured in the absence of substrate and in the presence of 1.2 µM $G6PDG_{50}$ or 350 µM NR. Data are shown as mean ± s.d. of n ≥ 3 independent experiments.

| | ATPase Activity (µmol ATP · min$^{-1}$) | | | | | |
|---|---|---|---|---|---|---|
| | **ClpB$_{WT}$** | **ClpB$_{T1}$** | **ClpB$_{T2}$** | **ClpB$_{N1}$** | **ClpB$_{N2}$** | **KJE** |
| **No substrate** | 2.06 ± 0.06 | 1.37 ± 0.07 | 7.66 ± 0.04 | 0.61 ± 0.01 | 1.54 ± 0.06 | 3.18 ± 0.19 |
| **G6PDH$_{50}$** | 1.92 ± 0.29 | 1.23 ± 0.13 | 7.72 ± 0.79 | 0.44 ± 0.02 | 1.55 ± 0.01 | 3.04 ± 0.09 |
| | **ClpB$_{WT}$ + KJE** | **ClpB$_{T1}$ + KJE** | **ClpB$_{T2}$ + KJE** | **ClpB$_{N1}$ + KJE** | **ClpB$_{N2}$ + KJE** | |
| **No substrate** | 6.27 ± 0.33 | 5.38 ± 0.09 | 13.69 ± 0.73 | 5.18 ± 0.51 | 5.44 ± 0.22 | |
| **G6PDH$_{50}$** | 12.61 ± 1.93 | 7.36 ± 0.09 | 14.27 ± 0.24 | 9.63 ± 1.09 | 5.24 ± 0.51 | |

**Table S4**. ATPase activities of wt ClpB and the different NBD ClpB variants in the absence and presence of the DnaK system and 1.2 µM G6PDG$_{50}$. Data are presented as mean ± s.d. of at least three independent experiments.
